# Performance of Working Memory Updating in Socially Anxious Individuals

**DOI:** 10.1155/2024/1799948

**Published:** 2024-03-20

**Authors:** Jing Yuan, Xiran Sun, Qin Zhang, Lixia Cui

**Affiliations:** ^1^School of Nursing, Hebei University, Baoding, China; ^2^Beijing Key Laboratory of Learning and Cognition and School of Psychology, Capital Normal University, Beijing, China; ^3^Yanjing Medical College, Capital Medical University, Beijing, China

## Abstract

Working memory updating plays a critical role in executive function. Few studies explored the working memory updating in socially anxious individuals. In this study, we wanted to explore the working memory updating in socially anxious individuals. We studied this issue by instructing participants to perform an emotional 2-back task, and recording their response time and accuracy. We found that high socially anxious individuals showed significant longer response time in positive word condition than that of negative and neutral words. But there was no significant difference in word type in low socially anxious group. In accuracy, we did not observe any significant difference in group, word type, and their interaction. These results indicate that socially anxious individuals have deficits in positive content updating, which have an important implication for developing method to reduce social anxiety.

## 1. Introduction

Socially anxious individuals have intense fear or anxiety about social interaction, social performance, or evaluation by others [1]. Cognitive theory suggests that bias in processing information related to social threat plays an important role in the pathogenesis and maintenance of social anxiety disorder [2].

Many studies have shown that socially anxious individuals have attentional bias towards threatening stimuli, using various experimental paradigms and materials [3–5]. This attentional bias may be manifested as hypervigilance to social threat, difficulty in disengagement, or attentional avoidance.

Interpretation bias is also an important manifestation of cognitive bias in socially anxious individuals [6]. Empirical studies have shown that socially anxious individuals interpret ambiguous social situations negatively or threateningly, and mildly negative social situations as disastrous [7, 8].

With regard to memory bias, there are relatively few studies on memory bias in socially anxious individuals. Previous studies have questioned whether memory bias is one of the characteristics of social anxiety [9]. However, recently, more and more studies have found that socially anxious individuals have memory bias in both explicit and implicit memory [10–12].

In addition to the cognitive bias towards negative stimuli, in recent years, researchers have begun to pay attention to how socially anxious people process positive stimuli and have proposed bivalent fear of evaluation model [13–15]. They think that fear of evaluation is important for social anxiety, including fear of both positive and negative evaluations. In empirical research, it is also found that people with social anxiety avoid eye contact with both the positive and negative video clips [16] and reduce willingness to approach genuine smilers [17].

In order to understand the specific information processing deficits in social anxiety, it is important to explore the mechanism leading to cognitive biases in social anxiety. Many researchers have focused on the executive function of anxious individuals [18, 19]. Using latent variable analysis, Miyake et al. [20] found that executive function consisted of three basic processes: (1) shifting between tasks or mental sets, (2) inhibition of dominant or prepotent responses, and (3) updating and monitoring the working memory contents (WM). Attentional control theory developed by Eysenck et al. [21, 22] suggests that anxiety impairs functioning of the goal-directed attentional system and increases the influence of the stimulus-driven attentional system. Adverse effects of anxiety on processing efficiency depend on two central executive functions involving attentional control: inhibition and shifting.

As we all know, WM has a limited capacity of about 3-4 items [23], which leads to the fact that although we are surrounded by a large amount of emotional information every day, only a small portion of it enters our processing. This limitation allows us to attend only selectively in relevant information [24]. Therefore, the ability to WM updating is crucial. WM updating is a complex capability that consists of multiple subprocesses to ensure that task-relevant representations enter WM, while irrelevant information is filtered out [20, 25]. People differ in their ability of WM updating. For example, compared with young people, elderly people have specific difficulty in updating information in WM [26, 27].

Besides age-related differences, individual differences in WM updating are correlated with psychopathology [28–30]. For instance, interference from task-irrelevant negative material might be a key mechanism of potentially intrusive ruminative thoughts in depression [31]. To be specific, great difficulty in removing task-irrelevant negative material is associated with the tendency to ruminate. Gustavson and Miyake [32] also confirmed the relationship between trait worry and WM updating. Trait worry is a tendency to have repeated negative thoughts about anticipated future events that are difficult to remove from the mind. They instructed participants to perform a WM updating task and demonstrated that levels of trait worry were not related to word-span performance but were related to performance on trials that required participants to effectively update WM.

Repetitive negative thinking is a transdiagnostic phenomenon that is present across affective disorders [33]. Negative rumination is also prominent in social anxiety disorders [34]. High socially anxious individuals tend to focus on negative information about themselves and how others perceive them in social settings, repeatedly comparing it to unrealistically high standards [35]. Chang et al. [36] found that compared to individuals with low ruminative response, individuals with high ruminative response spent less time removing outdated words from WM when the new to-be-remembered word was negative.

In one of the few studies on WM updating in socially anxious individuals, Segal et al. [37] measured WM updating of emotional content by socially anxious individuals. They instructed participants to perform an emotional 2-back task. That is, participants were asked to indicate whether a given face showing a certain emotion was the same as the emotion that was presented 2 trials before. Biases were assessed by intrusion cost in response times (RTs). Specifically, the term “intrusion trials” refers to the trials in which the stimulus presented on the screen differs from the one presented in trial N-2 (in a 2-back task) but is identical to trial N-1. They found that compared with the low socially anxious group, the high socially anxious group showed a diminished intrusion cost in irrelevant positive content. They suggested that high socially anxious individuals were better able to suppress positive stimuli than low socially anxious individuals. The study of Segal et al. [37] is the first study on the WM updating of socially anxious individuals. However, the effects reported by them are related not only to impaired WM updating but also to shifting between different emotional valence of stimuli [38]. To overcome this confusion, Zhang et al. [38] used the 2-back task with a block design (negative, neutral, and positive blocks) to examine the WM updating in depressed patients. There were 60 trials in each block, a total of three blocks, with half of them having “match” items and the other half having “mismatch” items. They found that compared to healthy controls, depressed patients performed poorer only when updating positive material. The block design was also used in an earlier study of WM updating in depressed patients [39]. They used neutral, sad, and happy faces as stimuli and found that compared with controls, depressed participants were both slower to disengage from sad stimuli and faster to disengage from happy facial expressions.

To recap briefly, WM updating plays an important role in understanding the mechanism of cognitive bias in socially anxious individuals. In the current study, we aimed to explore the WM updating in socially anxious individuals by an emotional 2-back task. According to Weeks et al.'s [13] opinion that fear of evaluation in general is important in social anxiety, we hypothesized the following: (1) High socially anxious (HSA) group would have deficits in WM updating in emotional words. However, based on the inconsistent results of previous empirical studies, it is difficult for us to predict the specific manifestation of WM updating deficits in socially anxious individuals. (2) There would be no difference in the three kinds of words in the low socially anxious (LSA) group.

## 2. Method

### 2.1. Participants

The sample size was calculated by GPower, on the one hand, and the parameters were set as effect size *f* = 0.25, *α* err prob = 0.05, and power (1 − *β* err prob) = 0.95. After calculation, the total sample size was 44. On the other hand, we referred to other works in the field, using similar sample sizes [31, 38, 40]. One hundred and fifty-three participants from a pool of undergraduate and graduate students at the university were screened via online Liebowitz Social Anxiety Scale (LSAS; [41]). According to Lv et al. [42], participants with scores greater than or equal to 60 were divided into the HSA group; according to He and Zhang [43], participants with scores of 38 or less were assigned to the LSA group. We telephoned participants who scored above 60 or below 38 on LSAS and invited them to participate in the experiment. Finally, 29 participants with high social anxiety (females = 26, *M*_age_ = 23.17, SD_age_ = 2.21; males = 3, *M*_age_ = 22.78, SD_age_ = 2.04) and 28 participants with low social anxiety (LSA) (females = 21, *M*_age_ = 23.79, SD_age_ = 2.01; males = 7, *M*_age_ = 22.66, SD_age_ = 2.33) voluntarily participated in the experiment. One participant in the HSA group was excluded from further analysis due to missing of WM updating recording. There was no significant difference in age between the HSA and LSA groups (*t* (55) = 1.10, *p* = 0.28). Participants also filled out the State-Trait Anxiety Inventory-State (STAI-S; [44]) before experiment. After the WM updating task, participants completed another two questionnaires—the State-Trait Anxiety Inventory-Trait (STAI-T; [45]) and Beck Depression Inventory-II (BDI-II; [46]). The scores of LSAS, STAI, and BDI-II in HSA and LSA groups are shown in Table 1. There were significant differences in STAI-T, STAI-S, and BDI-II between the HSA and LSA groups (see Table 1).

All participants were right-handed and had a normal or corrected-to-normal vision. The study was approved by the Research Ethics Committee of Capital Normal University (No. HDFYLL-KY-2022-007). All participants were given written informed consents and 40 ¥ as compensation.

Stimuli were presented on a 15.6⁣^″^ monitor (1366 × 768 pixels, 60 Hz refresh rate), with a black background, running by E-Prime software. Participants were seated in a comfortable chair in a dimly lit room at a 60 cm viewing distance.

### 2.2. Questionnaires

#### 2.2.1. Liebowitz Social Anxiety Scale

The Chinese version of LSAS [43] was employed to evaluate individual differences in social anxiety. LSAS consists of two subscales—a fear subscale and an avoidance subscale. Each subscale includes 24 items, listing socially relevant situations. The total score ranges from 0 to 144. The scale showed good psychometric qualities in terms of internal consistency and test–retest reliability [3].

#### 2.2.2. State-Trait Anxiety Inventory

The STAI is a self-report questionnaire composed of STAI-S and STAI-T two subscales, assessing the frequency and intensity of state anxiety and trait anxiety symptoms. The scale provides excellent psychometric properties with Cronbach's alpha as well as validity [47–49]. In the current study, the Cronbach *α* for the STAI-T was 0.916 and for the STAI-S was 0.942.

#### 2.2.3. Beck Depression Inventory-Revised

Depressive symptoms were assessed via the BDI-II, a 21-item self-rating measure for current depression severity. Reliability and validity of this scale were shown to be excellent in both clinical and nonclinical populations [49, 50]. In the current study, the Cronbach *α* for the BDI-II was 0.944.

### 2.3. Stimuli

A total of 60 adjectives, each consisting of two Chinese characters, were selected from the Chinese Affective Words System (CAWS, [51]) and based on valence, arousal, and familiarity. CAWS contains 1500 words, including 500 nouns, verbs, and adjectives, rated on a 9-Likert scale. Valence, also known as pleasantness, refers to an individual's subjective feelings of pleasure/unpleasantness. Arousal refers to the degree of bodily activation from calm to excitement generated by an individual's emotional experience. Familiarity refers to the reader's familiarity with a certain word or word, which is a subjective frequency and a comprehensive sensory experience of hearing, vision, and written form. The 60 words were evenly divided into three groups: negative words like “selfish” and “treacherous,” positive words like “smart” and “elegant,” and neutral words like “silence” and “euphemism.” There was a significant difference in the valence of the affective words (*M*_negative_ = 2.79, SD_negative_ = 0.16; *M*_positive_ = 7.10, SD_positive_ = 0.14; *M*_neutral_ = 4.86, SD_neutral_ = 0.66; *F* (2, 57) = 585.6, *p* < 0.001). In the arousal, the neutral words were significantly smaller than the positive and negative words (*M*_neutral_ = 4.31, SD_neutral_ = 0.56, *M*_positive_ = 5.15, SD_positive_ = 0.5, *M*_negative_ = 5.33, SD_negative_ = 0.62, *F* (2, 57) = 18.4, *p* < 0.001). But there was no significant difference in arousal between positive and negative words (*p* = 0.97). There was a significant difference in the familiarity (*F* (2, 57) = 28.30, *p* < 0.001). Pairwise comparison revealed significant differences in familiarity between stimuli with different valence (*M*_negative_ = 4.70, SD_negative_ = 0.40; *M*_neutral_ = 5.28, SD_neutral_ = 0.47; *M*_positive_ = 5.88, SD_positive_ = 0.60). There was no significant difference in the stroke counts (*M*_negative_ = 19.80, SD_negative_ = 6.48; *M*_neutral_ = 18.10, SD_neutral_ = 3.81; *M*_positive_ = 17.85, SD_positive_ = 5.15).

### 2.4. Procedures

**Figure 1:**
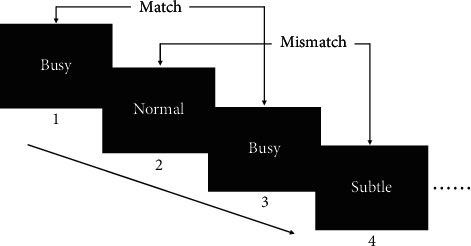
Illustration of the emotional 2-back task.

In the 2-back task, each trial started with a cross fixation being presented at the center of the screen for 450 ms, followed by the adjectives appearing for 500 ms. After the word disappeared, the participants were instructed to determine whether the word was the same as the word presented 2-back before and pressed corresponding buttons. Participants need to respond within 2000 ms. Once the participants pressed the response button, a 1500 ms of blank screen was presented. The first and second words were rendered without keystrokes, and the reaction time started from the third word. The procedure can be seen in Figure 1.

There were 3 blocks in the 2-back task, one block per emotional type. The order of the 3 blocks was counterbalanced between participants. Each block contained 60 trials, half of which were “match” and the other half were “mismatch.” Before the formal task, participants were instructed to complete a practice block with 30 trials. Unlike the formal task, practice block provided feedback. That is, after participants made judgments, feedback would appear, informing participants whether the response was correct or not. If the accuracy in the practice block reached more than 80%, participants can press the button to enter the formal experiment, otherwise, continue to practice.

### 2.5. Data Analysis

Most studies on socially anxious individuals or patients did not control trait anxiety because it is very difficult to differentiate trait anxiety from social anxiety [52, 53]. With regard to depression, we performed repeated measures ANCOVA, with group (HSA, LSA) as the between-subject factor, word type (negative, positive, neutral) as the within-subject factor, and depression as covariates. The results showed that there was no significant interaction effect between word type and depression in ACC (*F* (2, 98) = 1.44, *p* = 0.24) and RT (*F* (2, 98) = 1.03, *p* = 0.36). However, the interaction effect between word type and group was significant (*F* (2, 98) = 4.27, *p* = 0.17). Therefore, we preferred repeated measures ANOVA in this study.

The accuracy (ACC) and response time (RT) in 2-back tasks were analyzed using repeated measures ANOVAs, with group (HSA, LSA) as the between-subject factor and word type (negative, positive, neutral) as the within-subject factor. An alpha level of 0.05 was adopted as the critical value. If interactions were significant (*p* < 0.05), further simple effects would be analyzed with Bonferroni adjustments.

## 3. Results

The RT and ACC of HSA and LSA groups in three types of affective words are shown in Table 2. Five participants with ACC lower than 0.8 (3 participants in the HSA group, 2 participants in the LSA group) were excluded. If the response is incorrect, trial RT < 100 ms or RT > 3 SD (each participant under each affective word condition), the RT of the trial will be excluded when the RT index is analyzed. The proportion of the remaining trials was as follows: HSA group (negative: 93.0%; neutral: 93.5%; positive: 95.0%) and LSA group (negative: 95.5%; neutral: 97.0%; positive: 95.5%).

### 3.1. RT

**Figure 2:**
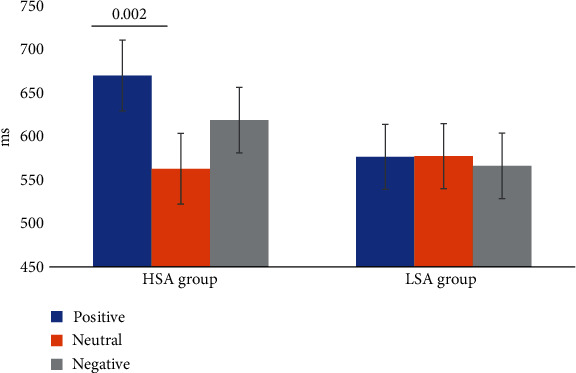
The mean reaction times of high and low socially anxious groups in different word types (the vertical bars extend and below the mean by one standard error of the mean).

We performed a 2 × 3 repeated measures ANOVAs on RTs. The results showed a significant main effect of word type, *F* (2, 100) = 3.252, *p* = 0.043, *η*_*p*_^2^ = 0.061, but the main effect of group was not significant (*p* = 0.375). We also observed a significant interaction of word type and group, *F* (2, 100) = 3.414, *p* = 0.037, *η_p_*^2^ = 0.064. Further simple effect analysis showed that in the HSA group, the RT of positive word was significantly slower than neutral word (*p* = 0.002). However, there was no significant difference between RT of positive word and negative word (*p* = 0.538), or RT of neutral word and negative word (*p* = 0.310). While in the LSA group, the significant difference between the three types of word in RT was not observed (*p* > 0.05) (see Figure 2).

### 3.2. ACC

We conducted 2 × 3 repeated measures ANOVAs on ACCs. There were no significant main effects of word type (*p* = 0.093), group (*p* = 0.096), and interaction of word type and group (*p* = 0.409).

## 4. Discussion

The purpose of this study was to investigate the characteristic of working memory updating of socially anxious individuals. To achieve this goal, we instructed high socially anxious and low socially anxious individuals to perform an emotional 2-back task to measure their WM updating. The results showed that in the HSA group, compared with negative and neutral words, participants showed slightly longer RT in updating positive word, but there was no significant difference in the RT of participants in the LSA group to update the three kinds of emotional words.

According to Ecker et al. [54], three subcomponents can be identified in WM updating, which are retrieval, transformation, and substitution. The various WM updating tasks used in the previous studies employ these three processes to varying degrees. The *n*-back task involves the retrieval component. Because one has to remember at least the last *n* items and retrieve the *n*th one back at every step. From this perspective, the findings in this study suggest that compared with neutral stimuli, socially anxious individuals take longer to retrieve positive stimuli, reflecting that socially anxious individuals are insensitive to positive stimuli. Similar evidence has been found in studies of facial expression recognition. Some studies have found that social anxiety moderates the recognition advantage for happy faces. To be specific, high socially anxious individuals have longer recognition RT for positive stimuli, compared with low socially anxious individuals [55]. In addition, previous studies have found that socially anxious individuals or social phobia have a lack of positive attentional bias [56, 57].

Both Segal et al.'s [37] study and our study found that people with high social anxiety had deficits in positive stimulus processing. However, we used different task setup from theirs. In their study, the faces with different emotional valences were presented in a random order across trials. They found a diminished intrusion cost in positive content in the high socially anxious individuals, which suggests a better inhibition for positive content in high socially anxious individuals. Instead, we used block design in this study (trials with an emotional valence in each block). We found that high socially anxious individuals showed significant longer response time in positive word condition than that of negative and neutral words, suggesting that they have deficits in positive content updating. Shifting and updating are two processes of the executive function [20]. Inhibition is a deliberate overriding of dominant or prepotent responses; updating is a constant monitoring and rapid addition/deletion of working memory contents [58]. These two executive processes, although moderately correlated with one another, are clearly separable.

The insensitive to positive stimuli plays an important role in anxiety maintenance [39]. Previous studies have put more attention to the processing of negative stimuli in socially anxious individuals, neglecting the processing of positive content. Our results suggest that socially anxious individuals ignore positive content in the environment, such as positive feedback, and therefore made their self-negative evaluations more deeply ingrained [59]. Hence, to reduce the social anxiety, avoidance of negative stimuli in external environment is not the only way. Training individuals to put their attention consciously on positive stimuli around them also helped reduce social anxiety [57]. Such as in attentional bias modification, positive-search training, a visual search task for searching a positive stimulus around by negative stimuli, seems more promising in modifying attentional bias in individuals with anxiety [60].

Although we found differences in how socially anxious individuals updated positive and neutral stimuli, we did not find differences in how they updated positive and negative stimuli. Our result may provide support for the bivalent fear of evaluation model [14]. This fear of positive and negative evaluation can be explained from a psychoevolutionary account of social anxiety [61, 62]. Socially anxious individuals perceive their environment as hierarchical and position themselves at the lower end of this ranking. The main goal is to stay in this hierarchy by avoiding upward and downward shifts. The purpose of fear of negative evaluation is to prevent further downward social mobility by avoiding further negative evaluation, while the primary function of positive evaluation fear is to prevent upward social mobility by avoiding positive evaluation.

However, we did not find any interaction influence of social anxiety and word type in ACC. Maybe the two indexes of RT and ACC may reflect different aspects of WM updating function. Eysenck and Calvo [63] put forward the processing efficiency theory to distinct effectiveness and efficiency. Effectiveness refers to the quality of task performance usually indexed by response accuracy. Efficiency refers to the relationship between the effectiveness of performance and the effort spent in task performance. They point out that negative effects of anxiety are predicted to be significantly greater on processing efficiency than on performance effectiveness, which is due to the compensation strategy. If auxiliary cognitive resources are available, impaired performance effectiveness is less likely to occur but at the cost of reduced efficiency. Visu-Petra et al. [64] supported this “compensation strategy.” They found that when simple verbal storage was required, high-anxious children showed only efficiency deficits; when higher verbal updating was required, the deficits in accuracy and efficiency were shown in high-anxious children.

One of the limitations of this study is that we did not find differences between positive and negative stimulus updating in socially anxious individuals, so we cannot draw a convincing conclusion about the deficits of positive stimulus processing in socially anxious individuals. This is well worth exploring in future research. It also should be noted that the participants in this study had subclinical social anxiety, so the generalization of the results is limited. When the participants are clinically anxious individuals, whether the compensation mechanism of efficiency and efficacy plays a role deserves further discussion. Another limitation is that in the current study, we focused on the valence of emotional stimuli and paid less attention to the arousal. However, as we know, emotional arousal also plays an important role in attention regulating and information processing. It has a different neural mechanism from emotional valence [65]. We can comprehensively explore the effect of emotion on WM updating from both of valence and arousal in the future study. The last but not the least, positive words are significantly more familiar than neutral words and negative words in this study. In our future research, we need to balance the familiarity of words.

In this study, we discussed the cognitive processing characteristics of socially anxious individuals. We found that socially anxious individuals showed a longer response time to WM updating of positive words, indicating positive information retrieval deficits. These results suggest that compared to instructing socially anxious individuals to avoid negative stimuli, we can put more attention to train them to focus on positive stimuli in the modification of social anxiety.

## Figures and Tables

**Table 1:** The scores of LSAS, STAI, and BDI-II in high socially anxious and low socially anxious groups.

	High socially anxious group (*n* = 29)		Low socially anxious group (*n* = 28)		*t* (55)	*p*
	*M*	SD	*M*	SD		
LSAS	78.59	18.26	22.75	11.26	13.84	⁣^∗∗^
STAI-T	49.24	9.54	38.11	7.15	4.97	⁣^∗∗^
STAI-S	49.24	10.62	37.36	8.10	4.74	⁣^∗∗^
BDI-II	13.79	11.75	4.68	6.17	3.65	⁣^∗∗∗^

Note: LSAS = Liebowitz Social Anxiety Scale; STAI-T = State-Trait Anxiety Inventory-Trait; STAI-S = State-Trait Anxiety Inventory-State; BDI-II = Beck Depression Inventory-II. ⁣^∗^*p* < 0.05; ⁣^∗∗^*p* < 0.01; ⁣^∗∗∗^*p* < 0.001.

**Table 2:** Reaction time and accuracy of high and low social anxiety groups in three types of affective words in the 2-back task.

	HSA group (*n* = 26)			LSA group (*n* = 26)		
	RT		ACC%	RT		ACC%
	*M*	SD		*M*	SD	
Positive words	669.84	212.99	84.95 (0.58)	576.35	201.47	83.49 (0.63)
Neutral words	562.65	180.52	88.20 (0.73)	577.36	200.23	83.95 (0.62)
Negative words	618.56	178.06	84.28 (0.90)	566.11	204.26	82.69 (0.78)

Note: RT = reaction time; ACC = accuracy rate.

## Data Availability

Data is available on request to the corresponding author.
